# Accurate and early prediction of the wound healing outcome of burn injuries using the wavelet Shannon entropy of terahertz time-domain waveforms

**DOI:** 10.1117/1.JBO.27.11.116001

**Published:** 2022-11-08

**Authors:** Mahmoud E. Khani, Omar B. Osman, Zachery B. Harris, Andrew Chen, Juin W. Zhou, Adam J. Singer, Mohammad Hassan Arbab

**Affiliations:** a Stony Brook University, Department of Biomedical Engineering, Stony Brook, New York, United States; b Renaissance School of Medicine at Stony Brook University, Department of Emergency Medicine, Stony Brook, New York, United States

**Keywords:** terahertz time-domain spectroscopy, burn assessment, terahertz spectroscopic imaging, machine learning, wavelet packet transform, Shannon entropy

## Abstract

**Significance:**

Severe burn injuries cause significant hypermetabolic alterations that are highly dynamic, hard to predict, and require acute and critical care. The clinical assessments of the severity of burn injuries are highly subjective and have consistently been reported to be inaccurate. Therefore, the utilization of other imaging modalities is crucial to reaching an objective and accurate burn assessment modality.

**Aim:**

We describe a non-invasive technique using terahertz time-domain spectroscopy (THz-TDS) and the wavelet packet Shannon entropy to automatically estimate the burn depth and predict the wound healing outcome of thermal burn injuries.

**Approach:**

We created 40 burn injuries of different severity grades in two porcine models using scald and contact methods of infliction. We used our THz portable handheld spectral reflection (PHASR) scanner to obtain the *in vivo* THz-TDS images. We used the energy to Shannon entropy ratio of the wavelet packet coefficients of the THz-TDS waveforms on day 0 to create supervised support vector machine (SVM) classification models. Histological assessments of the burn biopsies serve as the ground truth.

**Results:**

We achieved an accuracy rate of 94.7% in predicting the wound healing outcome, as determined by histological measurement of the re-epithelialization rate on day 28 post-burn induction, using the THz-TDS measurements obtained on day 0. Furthermore, we report the accuracy rates of 89%, 87.1%, and 87.6% in automatic diagnosis of the superficial partial-thickness, deep partial-thickness, and full-thickness burns, respectively, using a multiclass SVM model.

**Conclusions:**

The THz PHASR scanner promises a robust, high-speed, and accurate diagnostic modality to improve the clinical triage of burns and their management.

## 1 Introduction

According to the World Health Organization (WHO), 11 million burn injuries occur annually worldwide, 180,000 of which are fatal, with the vast majority occurring in low- and middle-income countries.^1^^,^^2^ Burn injuries result in significant skin necrosis and deep tissue damage.^3^ Burns created with different sources, such as scald, electrical, or flame injuries, invoke disparate pathophysiological responses, which necessitates using different treatment approaches.^4^ However, severe burns, regardless of their cause, lead to a highly dysregulated inflammatory response.^5^ The inflammatory response initiates tissue repair and the overall wound healing process to restore the skin barrier and the pliability and functionality of the skin.^6^^,^^7^ On the other hand, when the inflammatory cascade is triggered, it can also destroy the host tissue and contribute to organ failure.^8^ Moreover, wound healing is usually accompanied by the formation of abnormal, disfiguring lesions termed hypertrophic or keloid scars, which can lead to significant functional and social impairments.^9^^,^^10^ Early treatment of deep burns in the form of excision and grafting is critical for improving the healing process and reducing the infection rate to avoid excessive scarring.^7^^,^^11^

The initial assessment of the severity of a burn injury is paramount because it forms the basis for all subsequent triage and treatment plans.^12^ Superficial (S) and superficial partial-thickness (SPT) burns only affect the epidermis and the papillary dermis.^13^^,^^14^ They result in pain, weeping, and blisters and usually do not require surgical intervention. Deep partial-thickness (DPT) burns extend into the reticular dermis, partially destructing the dermal appendages, and may require surgery to promote an earlier healing and avoid infection and scarring. Full-thickness (FT) burns destroy the entire dermis and parts of the hypodermis. They result in significant necrotic tissue, are prone to infection, and require prompt surgical intervention. Estimating the burn depth by clinical evaluation, which involves visual and tactile examinations, is highly subjective and has consistently been inaccurate despite standardization attempts.^15^ Furthermore, determination of which burn wounds will heal spontaneously and which will benefit from an early excision and grafting can be challenging based on the initial examinations.^16^ Given the unreliability of estimation of burn depth by clinicians, the development of technological solutions to achieve an objective and accurate diagnosis is essential.^17^ Different modalities, such as laser Doppler imaging,^18^^,^^19^ spatial frequency domain imaging,^20^^,^^21^ infrared spectroscopy,^22^^,^^23^ infrared thermography,^24^ harmonic ultrasound imaging,^25^ and optical coherence tomography,^26^ have been utilized in preclinical and limited clinical trials to address this need. However, significant drawbacks such as a limited penetration depth, a limited field-of-view, a long acquisition time, and the overall cost have hindered the widespread use of these technologies.^27^

Over the past two decades, terahertz time-domain spectroscopy (THz-TDS) has emerged as a promising technique for the non-invasive sensing of various biological tissues.^28^^,^^29^ THz-TDS has been effective in the delineation of breast or skin cancer margins,^30^^–^^36^ diagnosis of brain,^37^ colon,^38^ and gastric tumors,^39^ screening diabetic foot syndrome,^40^^,^^41^ and monitoring corneal hydration for diagnosing glaucoma.^42^^–^^49^ In skin assessment applications, THz-TDS has been used for quantifying hydration changes in the skin.^50^^–^^55^ In addition, the efficacy of theoretical models at describing the interaction of THz radiation with living skin has been explored.^56^^–^^58^ In monitoring cutaneous burns and scars using THz-TDS, it has been shown that the THz reflectivity of FT burns and hypertrophic scars is higher compared with the normal skin.^59^^–^^61^ This increase in the THz reflectivity has been explained by the formation of post-burn interstitial edema.^62^^,^^63^ A combination of empirical parameters obtained by the THz-TDS measurements, such as the spectral slope and the area under the THz reflectivity curve, has been used to differentiate between superficial and deep burns in rodent and porcine burn models.^64^^,^^65^ Recently, we designed and fabricated a fiber-coupled THz spectral imager, named portable handheld spectral reflection (PHASR) scanner,^66^ to enable *in vivo* biomedical imaging applications using the THz-TDS technique. We showed that hyperspectral parameters obtained using the PHASR scanner can be utilized to longitudinally monitor the burn inflammatory process over a four-day post burn period.^67^ Moreover, we implemented deep learning^67^ and machine learning algorithms^68^ to automatically classify burn injuries into different severity groups using the Fourier-domain THz spectra obtained by the PHASR scanner.

In this work, we present a novel feature extraction approach to enhance the accuracy rate of diagnosis of *in vivo* burn injuries using the PHASR scanner. This technique utilizes the maximal overlap discrete wavelet packet transform (MODWPT) to obtain a multi-scale spectral decomposition of the THz-TDS waveforms measured ∼1-h post-burn (day 0). Additionally, the energy to Shannon entropy ratio (ESER) of MODWPT coefficients is calculated as the features for machine learning to automatically predict the burn early wound closure, as determined by histological assessment of the re-epithelialization rate 28 days after the burn induction (day 28). We achieved an accuracy rate of 94.7% in predicting the wound healing outcome using a binary Gaussian support vector machine (SVM) model. The high accuracy of this predictive modeling is highly valuable to timely distinguishing the burns that require surgical intervention from those that will heal spontaneously. Furthermore, we obtained the accuracy rates of 89%, 87.1%, and 87.6% in automatic diagnosis of SPT, DPT, and FT burns, respectively, using this technique. Our results confirm the utility of the PHASR scanner measurements in addition to novel feature extraction and machine learning techniques to yield an accurate and timely assessment of the burn injuries *in vivo*.

## 2 Method

### 2.1 Burn Protocol

**Fig. 1:**
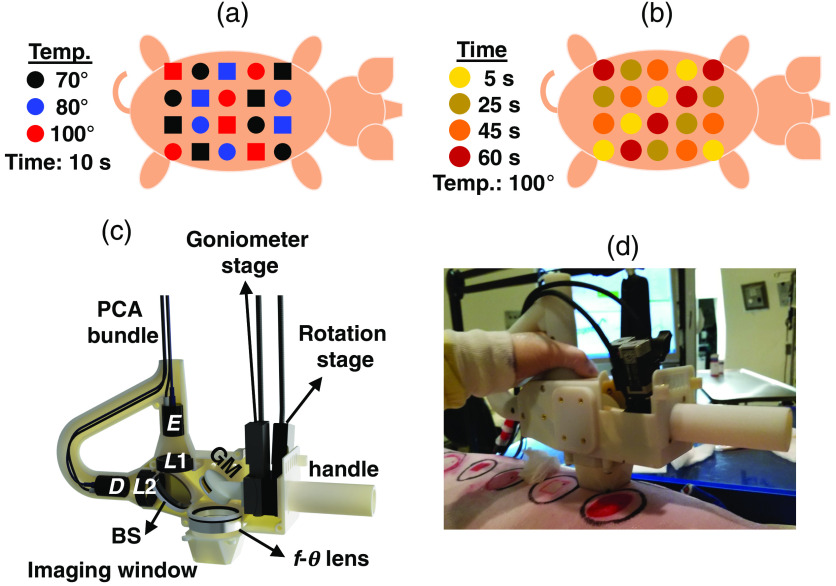
(a) The burn induction pattern on the dorsum in the first model. The burn locations created by scald and contact etiologies are shown by circle and square shapes, respectively. In this model, the exposure time is kept constant at 10 s, while the temperature is varied between 70°C, 80°C, and 100°C. (b) The burn induction pattern on the dorsum in the second model. The burns are created using the scald etiology. In this model, the temperature is kept constant at 100°C, while the exposure time is varied between 5, 25, 45, and 60 s. (c) The schematic of the optical components inside the PHASR scanner. This device incorporates a dual-fiber-laser spectrometer into a collocated, telecentric imaging configuration, which utilizes an f-θ lens and a two-axis motorized scanning system. (d) The PHASR scanner is shown as it is operated in the porcine imaging study.

The protocol of our animal study is reviewed and approved by the Institutional Animal Care and Use Committee at Stony Brook University. The *in vivo* experiments are conducted on two Landrace pigs because the porcine skin is physiologically and anatomically similar to the human skin.^69^ A total of 20 burns are created on the dorsum of each pig, resulting in 40 burn sites that are assumed to be independent in this study. Burns are created using two standard etiologies, including a metallic brass bar and a hot water scald, which represent the causes of many real-world clinical burn injuries.^70^^,^^71^ The burn induction procedure is described in detail elsewhere.^72^ Importantly, the diameter of each burn is ∼1  in., and all adjacent burns are 4-cm apart in the horizontal and vertical directions. The locations of the burns are uniformly distributed, following the patterns given in Figs. 1(a) and 1(b), to account for the anatomical variations of the cutaneous layers. We use various temperatures and exposure times to create different burn conditions. Burns of the first pig shown in Fig. 1(a) are created at a fixed exposure time of 10 s, while the temperature changes between 70°C, 80°C, and 100°C. Burns of the second pig shown in Fig. 1(b) are created at a fixed temperature of 100°C, while the exposure time varies between 5, 10, 25, 45, and 60 s. The dermal burn percentage on day 0 is obtained by the histological assessment of 4- or 8-mm punch biopsies stained with hematoxylin & eosin (H&E). These biopsies were collected approximately within 1 h of the burn induction. The burn depth is assessed by measuring the deepest point of injury, which is characterized by microvascular occlusion, collagen discoloration, or necrosis of follicular, mesenchymal, and adipocyte cells.^73^ Moreover, the re-epithelialization rate of the burns on day 28 post-burn is assessed histologically to determine the final wound healing outcome. For predicting the healing outcome using the THz spectra, we aim to classify the burns into fully re-epithelialized (FR) and none- or partially re-epithelialized (NPR) categories. Burns with a 100% re-epithelialization rate on day 28 are considered the FR group, and all other burns form the NPR category. For the automatic diagnosis of the severity group of each burn, we aim to categorically classify the burns into SPT, DPT, and FT groups. Accordingly, burns with <60% dermal burn depth are grouped in the SPT category. Burns with dermal burn depth in the 60% to 90% range are placed in the DPT burns. Burns with >90% depth of damage to the dermis are grouped as the FT burns. Although each burn site is labeled based on the histological assessment of one biopsy section, the scald and contact devices fabricated for the induction of the burn injuries are designed following the standardized approaches,^74^^,^^75^ which have been demonstrated to result in highly consistent and fairly homogeneous burns.

### 2.2 PHASR Scanner

We use the PHASR scanner to obtain the *in vivo* THz-TDS measurements within 1 h after the induction of the burn injuries.^66^ In this device, a dual-fiber-laser spectrometer (Menlo Systems, Inc., Newton, New Jersey) is incorporated into a collocated telecentric imaging configuration. The telecentricity is achieved by utilizing a f-θ lens^76^ and a two-axis motorized scanning system.^77^ Figure 1(c) shows the schematic of the optical components inside the PHASR scanner. THz pulses are generated by optical excitation of a photoconductive antenna (PCA) using the 1560-nm pulses of a femtosecond laser at a repetition rate of $f_{\mathrm{rep}}=100\;\mathrm{MHz}$. The generated beams are collimated using a TPX lens (L1) with a 50-mm focal length (Menlo Systems, Inc., Newton, New Jersey). A high-resistivity silicon beam splitter (BS) routs the collimated beams toward a gimbal mirror (GM). The GM is mounted on a two-axis motorized heliostat, which is composed of a goniometer and a rotational stage to raster scan the beam across the f-θ lens. The f-θ lens has a focal length of 40 mm and is made from high-density polyethylene. Importantly, this lens is custom designed such that a collimated beam passing through the front focus at a deflection angle of θ is focused at a distance of f×θ from the lens optical axis. Consequently, the focus is always parallel to the optical axis, and the spot size has a fixed value at the focal plane. Furthermore, because of the collocated design, reflections from the tissue retrace the path of the incident beam back to the BS, and a second TPX lens (L2) focuses them on the PCA inside the detector (D). The probe pulses generated by a second femtosecond laser at a repetition rate of $f_{\mathrm{rep}}-\Delta f$ asynchronously sample the THz electric field at the detector. In the asynchronous optical sampling (ASOPS) technique, Δf has a fixed value.^78^ For example, setting Δf=100  Hz results in a pulse acquisition time of 10 ms. By contrast, in the electronically controlled optical sampling (ECOPS) system, the value of Δf is varied periodically,^79^ which yields a much faster pulse acquisition time of only 0.5 ms. In this work, we incorporate both of these THz-TDS sampling mechanisms in the PHASR scanner. For the ECOPS measurements, a few reference measurements are calibrated against the ASOPS measurements of the same reference target to obtain a time-axis correction formula, which is described in detail elsewhere.^80^ The results of the first burn model shown in Fig. 1(a) are obtained using the ASOPS system. Field-of-view of the PHASR scanner operating with the ASOPS technique is $27\times 27\;{\mathrm{mm}}^{2}$. The results of the second burn model shown in Fig. 1(b) are obtained using the ECOPS system. Field-of-view of the PHASR scanner operating with the ECOPS technique is $37\times 27\;{\mathrm{mm}}^{2}$. Moreover, the acquisition time of the ASOPS system is ∼3  min over the field-of-view (250-ms per pixel), whereas the acquisition time for the ECOPS system is only 35 s over a larger field-of-view (35-ms per pixel). It should be noted that although the burn protocols in the two models are slightly different, because the labels are assigned by the assessment of the biopsy samples, the algorithm is agnostic to the protocol of burn induction. Figure 1(d) shows the PHASR scanner placed on a porcine burn model. Table 1 summarizes the optical parameters of the femtosecond lasers and the generated THz pulses.

**Table 1:** The optical parameters of the femtosecond laser pulses and the THz pulses generated by the PHASR scanner.

Laser parameter	Value	THz parameter	Value
Central wavelength	1560 nm±20 nm	THz bandwidth	0.1 to 1 THz
Average power	<60 mW	THz power	<60 μW
Pulse energy	>1 nJ	Beam divergence	±12.5 deg
Pulse width	<90 fs	THz pulse width	2 ps
Polarization	Linear	Polarization	Linear

### 2.3 Signal Conditioning

The data set comprises measurements from forty burns and eight healthy-skin (H) sites. At each location, a field-of-view of $27\times 27\;{\mathrm{mm}}^{2}$ for one pig and $37\times 27\;{\mathrm{mm}}^{2}$ for the other one is raster-scanned at a $1\text{-}{\mathrm{mm}}^{2}$ pixel size. We band-pass filter all measured electric fields at 0.1- to 1-THz passband. Each signal is composed of multiple reflections created at the interfaces of air, imaging window, and tissue in addition to a Fabry–Perot reflection pulse. The appearance of additional Fabry–Perot reflection pulses or a reflection phase shift could reveal if there was an air-gap between the imaging window and the tissue because of the lack of a good contact. Therefore, a measurement should be repeated while slightly more pressure was applied by the operator of the scanner to ensure that there was a perfect contact between the scanner and the burned lesion. The measurements at each burn site are aligned using the air-imaging window reflections, accounting for small mechanical drifts in the system. Afterward, we use a Blackman window to separate the imaging window-tissue reflections at a 25-ps time width around the peak amplitude of the main THz pulse. Following that, we identify and remove the pixels associated with the biopsies using the approach described in Ref. 68. Previously, we showed that the Mie scattering can produce significant spectral artifacts in the THz-TDS measurements.^81^ Various experimental designs and signal processing techniques to retrieve the THz spectroscopic information in the presence of scattering have been proposed in the literature.^82^^–^^86^ Here, we implement the spatial averaging approach to mitigate the artifacts caused by the skin appendages and the rough surface scattering. Therefore, we select all 5×5-pixel regions of interest (ROI) over the field-of-view of each burn or healthy measurement site and use the average of 25 pixels at each ROI as a single observation. As a result, there are 300, 332, 224, and 569 observations from the H, SPT, DPT, and FT categories, respectively.

### 2.4 Feature Extraction

#### 2.4.1 Maximal overlap discrete wavelet packet transform

There are no characteristic absorption lines in the THz spectra of biological tissues. Therefore, utilizing numerical feature extraction techniques is critical to achieving an automatic differentiation between the THz spectra of various pathologies. Wavelet transform has been used extensively for feature extraction in signal and image processing applications.^87^ Wavelet transform decomposes a signal into a set of scaling and wavelet coefficients. Each set describes the localized variations in the signal at a specific scale. The scaling coefficients represent the weighted averages of the signal over those scales, i.e., they have a low-pass filtering behavior. Conversely, the wavelet coefficients corresponding to the differences of those averages demonstrate high-pass filtering characteristics. The scale of decomposition dictates the intervals over which these averages are calculated. In discrete wavelet transform (DWT), the scale parameter increments dyadically over increasing levels of decomposition, which splits the spectral content of a signal into octave sub-bands.^87^ In addition, the wavelet and scaling coefficients are down-sampled by two at each decomposition level. Recently, we have shown that the down-sampling operation in DWT can interfere with accurate feature extraction in THz-TDS.^88^ In contrast, maximal overlap DWT (MODWT) does not incorporate the down-sampling operation, i.e., it has a constant time resolution at all levels of decomposition. Thus, the scaling and wavelet coefficients at each level are the same length as the original signal. However, the scale parameter still increments dyadically in MODWT, reducing the spectral resolution at the higher frequencies. It has been demonstrated that splitting the spectral content of a signal into equal sub-bands, in contrast to octave sub-bands, provides a richer analysis of the high-frequency components, yielding higher classification accuracy rates.^89^^,^^90^ Therefore, in this work, we use the MODWPT for the multi-scale spectral decomposition of THz-TDS waveforms. Similar to MODWT, MODWPT benefits from a constant time resolution at all decomposition levels. In addition, at decomposition level j, MODWPT splits the spectrum into $2^{j}$ sub-bands of equal bandwidth, resulting in an identical spectral resolution over the low- and high-frequency content.

**Fig. 2:**
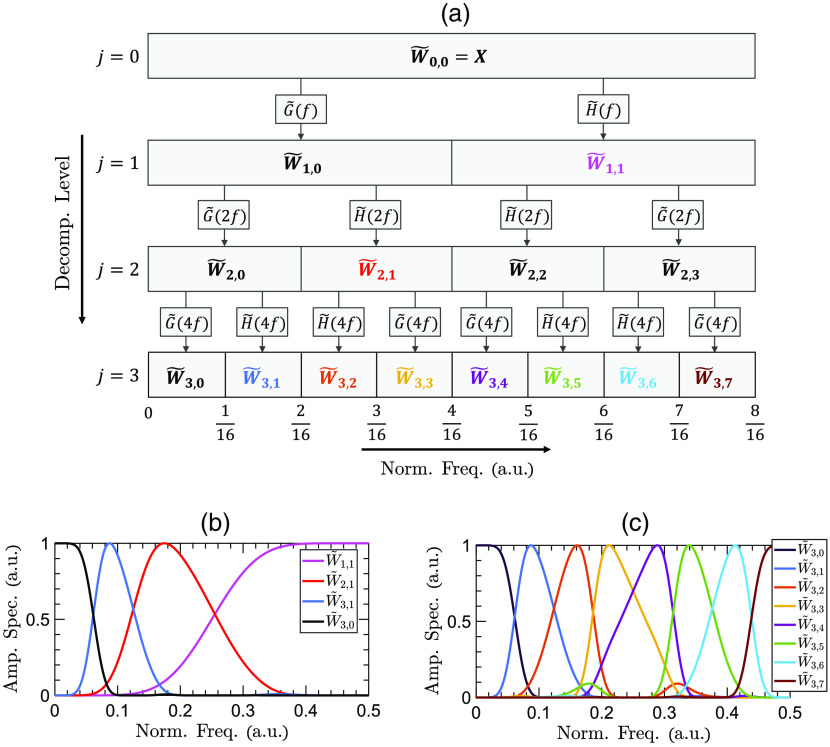
(a) The scale-frequency diagram of MODWPT. The first rectangle at level j=0 represents the entire spectrum of the original THz signal, X. At subsequent decomposition levels, the spectrum is divided into $2^{j}$ sub-bands of equal bandwidth by filtering the sub-bands of the previous stage with a pair of scaling and wavelet filters. The amplitude spectrum of the sub-bands are plotted for (b) MODWT and (c) MODWPT over j=3 levels of decomposition.

Figure 2(a) shows the scale-frequency diagram of MODWPT. In this diagram, the scale and normalized frequency parameters increase from top to bottom and left to right, respectively. The first rectangle at level j=0, labeled as ${\overset{˜}{W}}_{0,0}=X$, represents the entire spectrum of the original signal X. The subsequent two rectangles at level j=1 demonstrate that filtering X with a low-pass scaling filter, $\tilde{G}(f)$, and a high-pass wavelet filter, $\tilde{H}(f)$, divides the spectrum into two sub-bands of equal bandwidth. At the next level of decomposition, j=2, each of the sub-bands of level j=1 is separately filtered with the pair of scaling and wavelet filters, i.e., $\tilde{G}(2f)$ and $\tilde{H}(2f)$, respectively. Therefore, each sub-band is split into two new sub-bands of equal intervals. Subsequently, each of the sub-bands of level j=2 is divided equally at level j=3, and so on. Conversely, MODWT only retains the sub-bands ${\tilde{W}}_{1,1}$, ${\tilde{W}}_{2,1}$, ${\tilde{W}}_{3,0}$, and ${\tilde{W}}_{3,1}$. Figure 2(b) shows the amplitude spectrum of the sub-bands retained by MODWT. It can be noticed that the bandwidth of these sub-bands decreases dyadically with increasing the level of decomposition, providing different spectral resolutions over the various frequency components. In contrast, Fig. 2(c) shows that the eight sub-bands of MODWPT at the third level of decomposition have identical bandwidths, resulting in a similar spectral resolution over the various frequency components. The j’th level of MODWPT is calculated as^87^

$${\overset{˜}{W}}_{j,n}(t)=\underset{k}{\sum }\overset{˜}{u}(k){\overset{˜}{W}}_{j-1,\lfloor \frac{n}{2}\rfloor }(t-2^{j-1}k);\;n=0,1,\ldots ,2^{j}-1,$$ (1)

where

$$\overset{˜}{u}=\{\begin{array}{cc} \overset{˜}{g}, & \text{if }n\;\mathrm{mod}\;4=0\text{ or }3\\ \overset{˜}{h}, & \text{if }n\;\mathrm{mod}\;4=1\text{ or }2 \end{array},$$ (2)

where n is the number of the sub-band, increasing from left to right in Fig. 2(a), and $\left\lfloor \frac{n}{2}\right\rfloor$ is the integer part of $\frac{n}{2}$. It should be noted that MODWPT scaling and wavelet filters, $\tilde{g}$ and $\tilde{h}$, are related to DWT filters by $\tilde{g}=g/\sqrt{2}$ and $\tilde{h}=h/\sqrt{2}$, respectively, where g and h are derived from the same mother wavelet function.^91^ Also, the choice of the mother wavelet function and the number of decomposition levels affects the performance of the machine learning algorithms. In Sec. 2.5, we describe our approach to choosing a combination of the mother wavelet function and level of decomposition such that the cross-validation loss is minimized. We use the wavelet toolbox in MATLAB software (Mathworks, Natick, Massachusetts) to obtain the MODWPT coefficients.

#### 2.4.2 Energy to Shannon entropy ratio

The space of the MODWPT coefficients of the THz pulses over all measurements forms a high-dimensional data set. In particular, for a THz signal of length N, there are $2^{J}\times N$ MODWPT coefficients at the J’th level of decomposition. Therefore, further feature extraction is required to reduce the dimensionality. As a result, we first identify the MODWPT sub-bands associated with the measurement bandwidth following the scale-frequency diagram in Fig. 2(a). For example, the n=10 to n=80 sub-bands at the J=12th level of decomposition approximately correspond to the f=0.1−1  THz frequency range. Next, the Shannon entropy of the selected sub-bands is calculated to measure the amount of multi-scale information in the signal. Entropy is an important measure of information or uncertainty in a random variable. The Shannon entropy of a random variable x with possible outcomes of $x_{1},\ldots ,x_{n}$ occurring with probabilities of $P(x_{1}),\ldots ,P(x_{n})$ is given as

$$H(x)=-\overset{n}{\underset{i=1}{\sum }}P(x_{i})\log\;P(x_{i}).$$ (3)

We calculate the Shannon entropy of MODWPT coefficients following the approach described in Refs. 90 and 92. Accordingly, we find the Shannon entropy of each sub-band over the normalized energy of MODWPT coefficients at the final stage of the decomposition J, which is given as

$$H({\overset{˜}{W}}_{J,n})=-\underset{t}{\sum }P(t)\log(P(t));\;P(t)=\frac{{\left|{\overset{˜}{W}}_{J,n}(t)\right|}^{2}}{\underset{t}{\sum }{\left|{\overset{˜}{W}}_{J,n}(t)\right|}^{2}}.$$ (4)

Furthermore, we find the proportion of the energy of each sub-band with respect to the energy of its original signal X as

$$E({\overset{˜}{W}}_{J,n})=\frac{\underset{t}{\sum }{\left|{\overset{˜}{W}}_{J,n}(t)\right|}^{2}}{\underset{t}{\sum }{\left|X(t)\right|}^{2}}.$$ (5)

Finally, the ESER is calculated as

$${\mathrm{ESER}}_{n}=\frac{E({\tilde{W}}_{J,n})}{H({\tilde{W}}_{J,n})}.$$ (6)

Importantly, we normalize the ESER coefficients of the tissue measurements with those of the reference air measurements to deconvolve the system response. We use the deconvolved ESER coefficients as the predictors of the wound healing outcome or the burn severity group in the machine learning algorithms.

### 2.5 Machine Learning

**Fig. 3:**
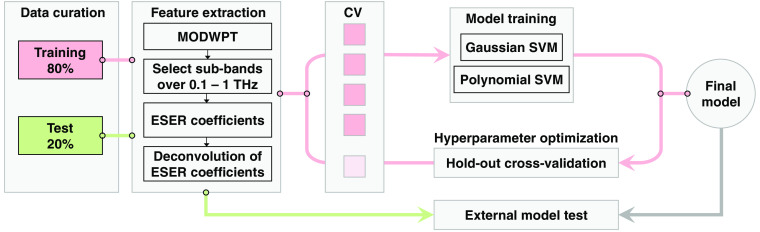
The machine learning pipeline. The measurements are split randomly into the training (80%) and test (20%) sets. After band-pass filtering the signals and separating the main THz-TDS pulse, the MODWPT coefficients are calculated. The MODWPT sub-bands corresponding to the measurement bandwidth of f=0.1−1  THz are selected. The ESER of selected sub-bands is calculated and normalized by the corresponding ESER of the reference air measurements to deconvolve the system response. These deconvolved ESER coefficients are used as the predictors. The dermal burn percentage on day 0 and the re-epithelialization rate on day 28 are used as the labels in training separate SVM classifiers. The hyperparameters of the classifiers, the choice of the mother wavelet function, and the level of decomposition of MODWPT are optimized over the five-fold cross-validation loss. The performance of the final trained model is evaluated over the 20% external test set. This process is iterated twenty times with the measurements being split randomly into the training and test sets at each iteration.

Figure 3 shows the machine learning pipeline. First, the pre-processed measurements are split randomly into the training (80%) and test (20%) sets. The data partitioning is performed at the ROI level. When evaluating the classifiers performance in Sec. 3, we show the mean and standard deviation of each parameter over twenty random iterations. Thereby, we verify that the model performance is not biased toward any specific configuration of training and test samples. As we described earlier, the deconvolved ESER of the MODWPT coefficients at the sub-bands associated with the bandwidth of the measurements is calculated as the predictor of the classification models. We have previously evaluated different machine learning classifiers, including SVM, linear discriminant analysis, an ensemble of decision trees (a random forest), and deep neural networks, for the classification of burn severity groups using THz-TDS.^68^^,^^72^ Among these, the SVM classifier is both effective and computationally efficient. The SVM algorithm has been employed for automatic classification of other pathological tissues, such as gastric,^39^ breast,^90^^,^^93^ and colon^94^ cancers, using the THz spectroscopy technique. Therefore, we also use the SVM model with a Gaussian or polynomial kernel function here. We train a binary SVM model to predict the healing outcome of the burns, i.e., to classify the burns into the FR and NPR groups. We also use the error-correcting output codes (ECOC) algorithm^95^ in MATLAB to train a multiclass model composed of four binary SVM classifiers to diagnose the burn severity group among the H, SPT, DPT, and FT categories. The hyperparameters used by the SVM and ECOC algorithms are chosen such that the five-fold cross-validation loss over the training set is minimized. These hyperparameters include the kernel scale, polynomial order (in the 2 to 20 range), box constraint, and multiclass coding (one-versus-one opposed to one-versus-all). In five-fold cross-validation, a model is first trained over 80% of the training set, and the remaining 20% is reserved for calculating the classification error.^96^ This process is iterated five times, with completely different samples being used as the validation set at each iteration. We also determine the mother wavelet function and level of decomposition to minimize the cross-validation loss. We choose the mother wavelet among the Daubechies wavelets with the maximal number of vanishing moments for a given support.^91^ The possible number of vanishing moments is in the range of 1 to 10, giving the mother wavelet functions db1, db2, …, db10. The number of decomposition levels is also selected from the range of 8 to 13. Using a level of decomposition >13 is computationally expensive, without a significant improvement in the accuracy rates. In Sec. 3, we present the performance of each classifier over the training, validation, and test sets. The validation set results present the outcome of the five-fold cross-validation over the training set, and the test set results are calculated over the 20% external test set. We calculate the sensitivity, specificity, and accuracy rates, in addition to the area under the receiver operating characteristic curve (ROC-AUC), of each model. The ROC curves are formed using the predicted probability assigned to each observation belonging to a burn severity or re-epithelialization group.^97^ A ROC curve shows the true positive rate versus the false positive rate at different thresholds selected over the predicted probabilities. A higher ROC-AUC indicates a better predictive performance by the model.^98^

## 3 Results

**Fig. 4:**
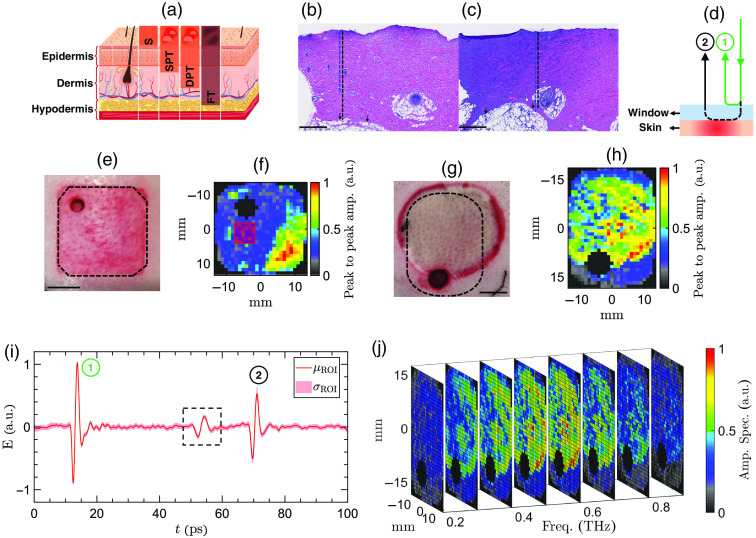
(a) The anatomy of the skin layers, composed of epidermis, dermis, and hypodermis. Burn injuries can be divided into superficial (S), SPT, DPT, and FT groups, depending on the extension of the dermal burn depth. (b), (c) Two example microscopic images of the biopsy slices (H&E stained), extracted from a DPT and an FT burn, respectively. The blue arrows point at damaged microvasculature or necrotic cells, and the black ones point at the full dermis margin. (d) A schematic of the measured reflection pulses in the PHASR scanner. (e), (f) The images of an example contact burn obtained using a digital camera and the PHASR scanner, respectively. The color axis represents the peak-to-peak amplitude of the time-domain THz reflections at the burn and imaging window interface. (g)–(h) Similar to (e) and (f) for an example scald burn. (i) The mean and standard deviation of the pulses of an ROI delineated by a red square in (f). (j) A sub-sampled representative 3D data cube composed of spectral images of the burn in (g). The color axis is the normalized amplitude spectrum obtained by the Fourier transform of the THz pulses.

Figure 4(a) shows the physiology of skin comprising epidermis, dermis, and hypodermis. It illustrates how the burn severity group corresponds to the percentage depth of the dermal burn. Figures 4(b) and 4(c) represent two examples of the histological assessment of the burn depth on day 0. They show the microscopic images of the biopsy slices obtained from a DPT and an FT burn, respectively. The scale bar is 500  μm at a 5× magnification ratio. In Figs. 4(b) and 4(c), the blue arrows point to microvascular damage or necrotic cells, and the black ones mark the boundary between dermis and hypodermis. The blue and black dashed lines show the burn depth and the thickness of the dermis. The depth of the deepest point of injury is divided by the thickness of the dermis to calculate the percentage depth of the dermal burn. Figure 4(d) shows a schematic of the measured reflection pulses in the PHASR scanner. These reflections originate from the interfaces between air, imaging window, and skin and are labeled as 1 and 2. Figure 4(e) shows the image of a contact burn obtained using a digital camera. The dashed black lines delineate the effective field-of-view of the PHASR scanner operating with the ASOPS system. An example of a 4-mm punch biopsy can be seen in the top left corner of the field-of-view. The scale bar in Fig. 4(e) is 1 cm. Figure 4(f) shows an example THz-TDS image of the burn in Fig. 4(e). The color axis represents the peak-to-peak amplitude of time-domain THz reflections at the burn and imaging window interface. The black area close to the center marks the pixels associated with the biopsy. These pixels are identified using the Fabry–Perot reflection pulses that appear after the second THz pulse. They originate from the air gap between the imaging window and the tissue in the biopsy section. The biopsy pixels are not included in the machine learning. The corner black pixels are also excluded because part of the beam is blocked by the housing in the corners. Figure 4(g) shows an example scald burn obtained using a digital camera. The dashed black lines delineate the effective field-of-view of the PHASR scanner operating with the ECOPS system. An 8-mm punch biopsy can be seen at the bottom of the field-of-view. Figure 4(h) shows an example THz-TDS image of the burn in Fig. 4(g). Similar to Fig. 4(f), the black areas mark the biopsy and corner pixels, which are excluded from the data set. Figure 4(i) shows the mean and standard deviation of the pulses of a single ROI. This ROI is delineated by a red square in Fig. 4(f). As we described in Sec. 2.3, the first reflection is used to align all measurements at each burn site. The pulse outlined by a dashed black rectangle represents an internal reflection caused by the BS. We isolate the imaging window-tissue reflections using a Blackman window within a 25-ps time width around the peak of the THz pulse. The MODWPT coefficients are calculated for the pulses averaged over the pixels of each ROI. Figure 4(j) shows a sub-sample of a three-dimensional (3D) data cube composed of the spectral images of the burn shown in Fig. 4(g). For the purpose of visualization, only images at f=0.2,0.3,…,0.9  THz are shown in Fig. 4(j). The color axis in Fig. 4(j) is the normalized amplitude spectrum obtained by the Fourier transform of the THz pulses. In contrast, the application of the MODWPT to THz signals results in a four-dimensional data set comprising two spatial directions in addition to the time and scale dimensions.

**Fig. 5:**
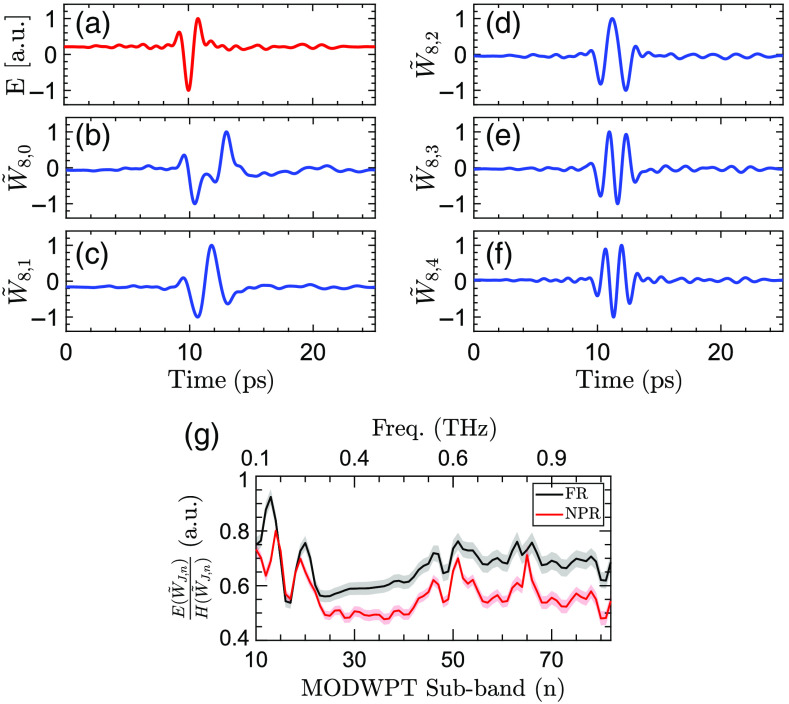
(a) The THz electric field measurement of a representative DPT burn. The first five MODWPT sub-bands, including (b) ${\tilde{W}}_{8,0}$; (c) ${\tilde{W}}_{8,1}$; (d) ${\tilde{W}}_{8,2}$; (e) ${\tilde{W}}_{8,3}$; and (f) ${\tilde{W}}_{8,4}$. The MODWPT coefficients are calculated using the db1 mother wavelet at the J=8th decomposition level. The wavelet coefficients of each sub-band are min–max normalized. (g) The ESER coefficients calculated for the n=10 to n=80 sub-bands, corresponding to the spectral range of f=0.1−1  THz, at the J=12th level of decomposition. The ESER of the burn measurements is normalized with the ESER of reference air measurements to deconvolve the system response. The black and red lines show the average and the 95% confidence interval of the ESER coefficients of all measurements belonging to the FR and NPR groups, respectively.

Figure 5 shows the MODWPT coefficients of an example THz-TDS pulse and the ESER coefficients of the FR and NPR burn groups. Figure 5(a) shows THz-TDS electric field measurement of a DPT burn. The MODWPT coefficients of the first five sub-bands at the J=8th decomposition level calculated using the db1 mother wavelet are shown in Figs. 5(b)–5(f). The passband of each sub-band can be obtained following the scale-frequency diagram of Fig. 2(a). For example, ${\tilde{W}}_{8,0}$ is formed by filtering ${\tilde{W}}_{7,0}$ using the up-sampled scaling filter $\tilde{G}(2^{7}f)$. It is approximately associated with the spectral content of the signal over f=0−0.2  THz. Similarly, ${\tilde{W}}_{8,1}$ shown in Fig. 5(c) is calculated by filtering ${\tilde{W}}_{7,0}$ with the up-sampled wavelet filter $\tilde{H}(2^{7}f)$. Therefore, its coefficients are associated with the spectral content of the electric field over f=0.2−0.39  THz, and so on. Figure 5(g) shows the deconvolved ESER coefficients corresponding to the frequency range of f=0.1−1  THz at the J=12th level of decomposition calculated using the db1 mother wavelet. The black and red lines show the average ESER coefficients of all measurements belonging to the FR and NPR groups, respectively. The error regions display the 95% confidence interval of the mean. It can be observed that an increase in the severity of the burns corresponds to a decrease in the ESER coefficients at most frequencies.

**Fig. 6:**
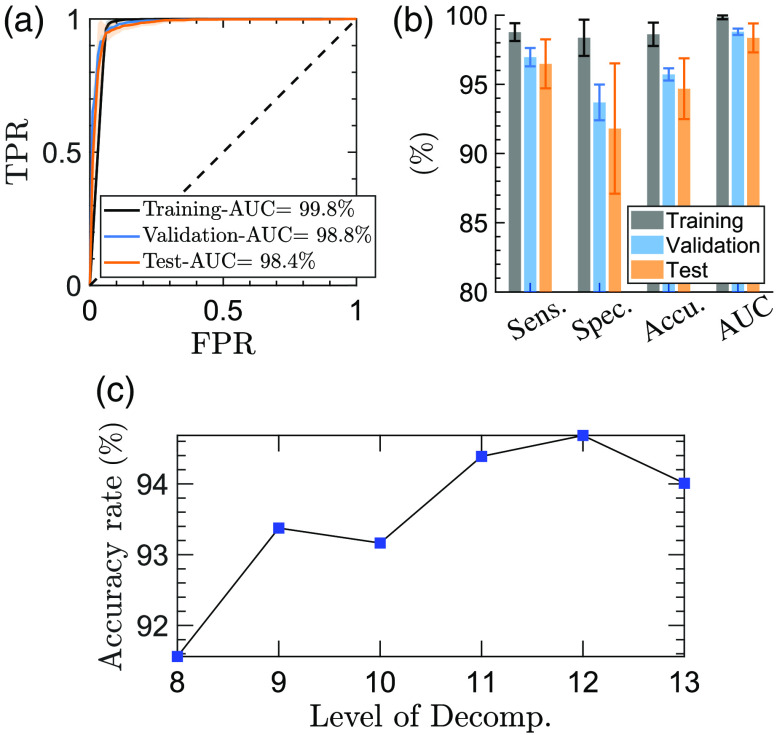
(a) The ROC curves obtained by predicting the re-epithelialization status of the burn injuries on day 28 using the THz-TDS measurements on day 0 in a binary SVM model. The dashed diagonal line shows the ROC curve of a random predictor. (b) The bar plot shows the sensitivity, specificity, accuracy, and ROC-AUC values over the training, validation, and test sets. The error regions in (a) and the error bars in (b) give the standard deviation of each parameter over twenty random iterations of the training and testing of the model. (c) The effect of the MODWPT decomposition level on the accuracy rate of diagnosing FR versus NPR burns using the db1 mother wavelet function.

Figure 6 shows the model performance in predicting the re-epithelialization status of the burns on day 28 using the ESER of the TH-TDS measurements on day 0. The ROC curves shown in Fig. 6(a) demonstrate average ROC-AUC values of 99.8%, 98.8%, and 98.4% over the training, validation, and test sets, respectively. The bar plots in Fig. 6(b) show the performance of the binary SVM model in terms of sensitivity, specificity, and accuracy rate. We achieved an average accuracy rate of 94.7% in the test set over the 20 random iterations. The corresponding sensitivity and specificity rates are 96.5% and 91.8%, respectively. The error bars and the error regions in Fig. 6 show the standard deviation over 20 random splittings of the measurements into the training and test sets. Figure 6(c) displays the effect of the MODWPT decomposition level on the accuracy rate of the SVM model in diagnosing FR versus NPR burns using the db1 mother wavelet function. The combination of db1 mother wavelet and J=12 level of decomposition yields the highest accuracy rate in the validation set. Therefore, a similar combination is used to calculate the ESER coefficients of the measurements in the external test set.

**Fig. 7:**
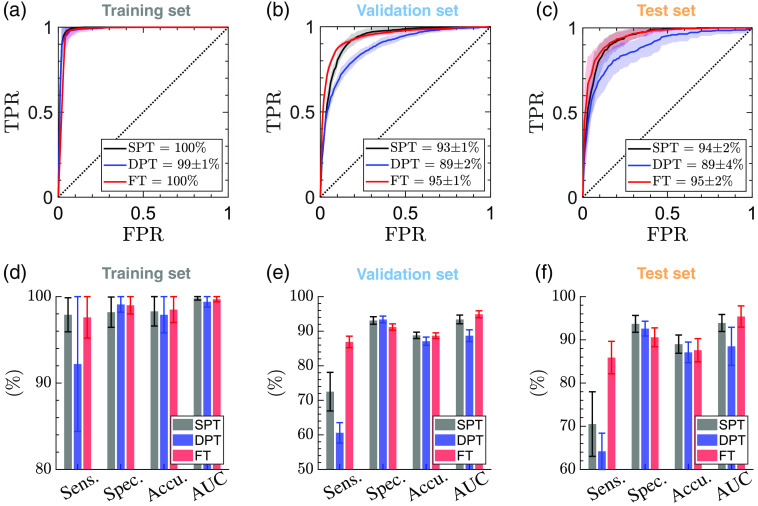
The ROC curves obtained by classification of the burn injuries into SPT, DPT, and FT groups over the (a) training; (b) validation; and (c) test sets using the ESER of the THz-TDS measurements in a multi-class SVM model. The ground truth of each burn’s severity grade is measured histologically on biopsies obtained on day 0. The bar plots present the sensitivity, specificity, accuracy, and ROC-AUC values over the (d) training; (e) validation; and (f) test sets. The error bars give the standard deviation of each parameter over 20 random iterations of training and testing the model.

Figure 7 shows the results of the automatic diagnosis of the severity group of the burns, as determined by independent histological assessment based on the percentage burn of the dermis on day 0. Figures 7(a)–7(c) show the ROC curves of the diagnosis of each burn group over the training, validation, and test sets. The average ROC-AUC values obtained for recognition of SPT, DPT, and FT burns in the test sets are 93.9%, 88.5%, and 95.4%, respectively. Figures 7(d)–7(f) provide the sensitivity, specificity, and accuracy rates in diagnosing the burn depth. The SPT, DPT, and FT burns in the test sets are identified with the average accuracy rates of 89%, 87.1%, and 87.6%, respectively. The corresponding specificity rates are 93.7%, 92.6%, and 90.6%, while the sensitivity rates are 70.5%, 64.2%, and 85.9%. Similar to Fig. 6, the error bars and the error regions in Fig. 7 show the standard deviation over 20 random splittings of the measurements into the training and test sets.

## 4 Discussion

Table 2 summarizes the performance of the models over the training, validation, and test sets for estimation of the burn depth on day 0 and predicting the wound healing outcome on day 28. It can be noticed that the THz-TDS measurements on day 0 can predict the wound healing with a higher accuracy rate of 94.7% compared with the diagnosis of the burn severity groups with the average accuracy rate of 87.9% for the SPT, DPT, and FT categories. It has been indicated in the literature that the biopsies obtained in the first 24 h post-burn period can underestimate the severity of the burns.^75^ This can be because of either the burn wound progression over the inflammatory cascade of the tissue or the inability of the H&E staining to reveal the functional cell damage.^75^ Therefore, the re-epithelialization rate of the burns obtained three or four weeks post-burn is a more reliable histological assessment to establish the ground truth of the burns requiring grafting or healing spontaneously. It also should be noted that predicting the wound healing outcome is a binary classification task. By contrast, estimation of the burn depth is a multiclass supervised machine learning problem (the number of classes is four in this work). Other depth of injury grading mechanisms, such as splitting the dermal burn depth into quarters from superficial to deep based on the depth of damage within the dermis, have also been suggested.^99^ Defining a multiclass modeling problem can result in a smaller number of observations within each group, affecting the accuracy, sensitivity, and specificity rates of diagnosis. Therefore, the accuracy rate of automatic burn depth estimation can be improved by adding more DPT samples to the data set. Furthermore, we achieved the higher specificity rates of 93.7%, 92.6%, and 90.6% in estimating the burn depth compared with the corresponding sensitivity rates of 70.5%, 64.2%, and 85.9%, respectively. The higher specificity of the model means that there are fewer false-positive diagnoses in each group, which is a desirable feature when a diagnostic test is aimed to recommend an invasive or costly procedure. Moreover, there were fewer DPT burns in our measurement set in comparison with the H, SPT, and FT groups, which can explain the lower sensitivity of the machine learning model to the DPT burns. Another limitation of the current implementation is that the dataset was partitioned at the ROI level to create the training and test sets, in addition to the data splitting for the five-fold cross validation of the training set. Although iterating this partitioning randomly can avoid the performance of the classifier to be biased toward a specific configuration of the measurements included in the training and test sets, there still might be a higher correlation between the ROIs within the same burn in comparison with the correlation between the ROIs from different burn sites. Partitioning the dataset at the burn level can prevent the correlation between the ROIs to affect the reported results. On the other hand, this form of data partitioning can be challenging for multi-group classification tasks, such as the classification of the skin tissues into H, SPT, DPT, and FT burns, for which the number of burn sites is limited and the distribution of the burns between different categories is highly unbalanced. Therefore, expanding the available observations set can enable partitioning of the dataset at the burn level, which is a more desirable form of data splitting.

**Table 2:** The summary of the performance of the SVM models in diagnosis of the burn severity groups and predicting the wound healing outcome based on the percentage of dermal burn on day 0 and the re-epithelialization rate on day 28, respectively.

		Sensitivity (%)	Specificity (%)	Accuracy (%)	ROC-AUC (%)
**SPT**	Training	97.9	98.2	98.3	99.8
	Validation	72.5	93.1	88.8	93.4
	Test	70.5	93.7	89	93.9
**DPT**	Training	92.2	99.1	97.9	99.4
	Validation	60.6	93.4	87.1	88.7
	Test	64.2	92.6	87.1	88.5
**FT**	Training	97.6	99	98.5	99.7
	Validation	86.9	91.2	88.7	94.9
	Test	85.9	90.6	87.6	95.4
**FR**	Training	98.8	98.4	98.6	99.8
	Validation	97	93.7	95.7	98.8
	Test	96.5	91.8	94.7	98.4

It has been reported that the blood perfusion unit measured using the LDI technique is the same between the burned and normal skin until ∼24h post-burn in the porcine models.^75^ Similarly, it has been proved that the blood perfusion in SPT and DPT burns is identical in the acute post-burn period, i.e., the first 48 h. The ability of the THz-TDS technique to identify the fully re-epithelialized burns only 1 h post-burn suggests the high potential of this technique for utilization in the acute burn assessment. The variations in the water content, chemical, and structural composition of the skin, e.g., changes in the cutaneous adnexal structures and biomolecules such as collagens and proteins, are the main sources of contrast between the THz reflectivity of the burns of different severities. However, isolation of the effect of each of these parameters requires further investigation. Furthermore, the classification accuracies obtained using the wavelet-domain features are superior to the results obtained using the Fourier amplitude spectra reported previously.^68^

## 5 Conclusion

We described a non-invasive technique utilizing the PHASR scanner and supervised machine learning to automatically estimate the severity of a burn injury and predict its wound healing outcome. Burn injuries of different severity grades, representing SPT, DPT, and FT wounds, were created by standardized scald and contact etiologies in two porcine models. We used the ESER of MODWPT coefficients of THz-TDS waveforms for *in vivo* burn assessment. We investigated the utility of the THz measurements obtained on day 0 to predict the wound healing outcome on day 28 for the first time. We achieved an average accuracy rate of 94.7% in predicting the wound healing outcome over the test set. We also reported specificity and sensitivity rates of 96.5% and 91.8% for this task. Additionally, the accuracy rates obtained using a multiclass SVM model to diagnose SPT, DPT, and FT burns were 89%, 87.1%, and 87.6%, respectively.

Similar to other automatic medical diagnosis applications, including more observations helps to validate and improve the reported accuracy, sensitivity, and specificity rates. Therefore, expanding the available experimental data set is warranted for future studies. Moreover, investigating the longitudinal variations in the ESER coefficients on a day-to-day basis and their correlation with the histology measurements can provide further insight into the utility of the PHASR scanner for monitoring the inflammatory cascades in burn injuries. Overall, the reported experimental results promise a robust, high-speed, and affordable diagnostic modality to improve the accuracy rate of non-invasive burn assessment in clinical settings. In addition, the demonstrated techniques can be utilized for studying other forms of lesions, ranging from various forms of cancerous tissues to skin burns caused by different sources. However, different imaging applications might require certain considerations to deal with artifacts caused by sources such as skin surface variations or lack of a contact between the imager and the tissue. Finally, incorporating time-resolved, polarimetric measurements^100^ in the future generations of the PHASR scanner, which can elucidate the polarization-sensitive signatures of skin, can potentially improve the diagnostic capabilities.

## Code, Data, and Materials Availability

Datasets and analysis codes used in this manuscript are available upon email request to Dr. Hassan Arbab at hassan.arbab@stonybrook.edu.
